# A Smart Sensing and Routing Mechanism for Wireless Sensor Networks

**DOI:** 10.3390/s20195720

**Published:** 2020-10-08

**Authors:** Li-Ling Hung

**Affiliations:** Computer Science and Information Engineering, Aletheia University, New Taipei 25103, Taiwan; llhung@au.edu.tw

**Keywords:** cooperative sensing, energy-efficient routing, monitoring accuracy, monitoring lifetime, mutual confirming

## Abstract

Wireless sensor networks (WSNs) have long been used for many applications. The efficiency of a WSN is subject to its monitoring accuracy and limited energy capacity. Thus, accurate detection and limited energy are two crucial problems for WSNs. Some studies have focused on building energy-efficient transmission mechanisms to extend monitoring lifetimes, and others have focused on building additional systems to support monitoring for enhanced accuracy. Herein, we propose a distributed cooperative mechanism where neighboring sensors mutually confirm event occurrences for improved monitoring accuracy. Moreover, the mechanism transmits events in a time- and energy-efficient manner by using smart antennae to extend monitoring lifetimes. The results of the simulations reveal that monitoring lifetime is extended and time for event notifications is shortened under the proposed mechanism. The evaluations also demonstrate that the monitoring accuracy of the proposed mechanism is much higher than that of other existing mechanisms.

## 1. Introduction

A wide range of applications have been proposed for wireless sensor networks (WSNs), including those in military, surveillance, environmental monitoring, and healthcare settings [1,2,3]. Internet of Things (IoT) is the technical basis of WSNs [4], and their application in this context has become more common in recent years; hence, topics surrounding WSNs have become increasingly noteworthy.

For WSNs, the objective of monitoring is to detect abnormalities, known as events. It is assumed that corresponding sinks should be notified of events: for WSNs, event detection and event notification are two key issues. Some researchers have proposed additional detecting systems to verify or further reveal event occurrence to improve event detection [5,6]. However, if sensors do not inform sinks of detected events, the monitoring efficiency of the WSN is poor. In general, this efficiency is an important factor when evaluating the performance of a monitoring WSN [7]. To improve the efficiency of event notification, researchers have proposed preventing interfering transmissions [8] because interference among transmissions may cause messages to not be received correctly. In general, a shorter routing path means that events can be detected efficiently because notifying the sink of the event requires short time and less energy. For monitoring the environment efficiently, studies also developed flexible mechanisms to adjust sensors’ occasions of triggering or relaying event notifications [9,10,11].

Because energy in WSNs is often limited to that stored by portable batteries, energy is one of the most crucial problems. The problem is discussed in two aspects: (1) harvesting energy from other equipment or environments [12,13], and (2) designing energy-efficient sensor mechanisms [14,15,16,17,18,19,20]. For harvesting energy, specific hardware can be designed and takes time to implement. Researchers have worked to resolve energy efficiency problems to prolong the monitoring lifetime of WSN applications [17,18,19,20]. In WSNs, energy consumption for transmitting is much greater than that for monitoring (or sensing) and receiving. If a sensor unconditionally forwards all received packets to sinks, because of wireless broadcasting, many redundant packets flow through the network; this is called a packet storm or broadcast storm. Packet storms waste a large amount of energy for redundant transmissions. Some researchers have proposed saving energy by avoiding duplicate transmissions at event notification [17,21,22,23]. The routing path for an event to its sink is formed by the sensors relaying it. The length of a routing path depends on the number of sensors relaying (or transmitting) the event. Moreover, a longer routing path requires more time and consumes more energy for transmissions. Some researchers have proposed reducing energy consumption in routing mechanisms [18,19,24,25].

Smart antennae improve the spatial diversity because they support simultaneous transmissions or simultaneous receptions of different messages in different directions [26,27]. Hence, smart antennae have the advantage of improving the transmission efficiency and avoiding interference of transmissions. Through arranging transmissions and receptions appropriately, the communication efficiency can be improved significantly. However, building the related hardware system also entails greater costs. Recently, the cost of new generation smart antenna has become much lower [28]. Therefore, the related hardware or applications can be developed more widely.

In this paper, we propose a smart sensing and routing mechanism (SSRM) for WSN applications. The mechanism employs a wide sensing range overlapping with monitoring regions in the environment. When an event occurs, more than two sensors mutually confirm the occurrence of events to avoid errors or false alarms. After confirmation, the event is transmitted to a corresponding sink rapidly and with relatively less interference. Furthermore, we also propose increasing the flexibility of the mechanism by allowing the monitoring parameters to be adjusted periodically. Because the sensors do not frequently transmit normal environment data, sensors’ transmission energies are mostly required for abnormal situations. The main contributions of this paper are as follows:

1. We propose transmissions using two antenna types for different targets: omnidirectional antennae for confirming events with one-step neighbors and smart antennae for transmitting events to two-step neighbors parallelly.
2. We propose a parallel transmission model for efficient transmissions using smart antennae.
3. We provide an adjusting model, allowing sensors to improve the flexibility of WSNs.

## 2. Related Studies

Because the energy available to WSNs is often limited by portable batteries, many studies have focused on improving energy efficiency for prolonging their monitoring lifetimes. To reduce energy consumption, Agarkhed [15] and Xu [16] classified events with different priorities. Event packets were sent according to their priorities. A low-priority packet may wait and aggregate with later high-priority packets. In addition, the energy consumption for transmissions was much more than that for sense and receipt. To prevent duplicate transmissions, Reddy [17] and Chhabra [18] proposed cluster-based mechanisms, whereby detected events were sent to sensors at the heads of clusters, and then the heads transmitted the events to corresponding sinks. These cluster-based transmission models avoided duplicate transmissions and reduced transmission times for event notifications, but cluster-based mechanisms consume energy for head selections. In addition, energy consumption by cluster heads is much greater and may require more transmissions if the detecting sensor is nearer to the sinks than its head. Thus, if the abilities and residual energies of sensors are similar, non-cluster-based mechanisms may be more appropriate. Our mechanism applies to environments monitored by sensors with similar abilities and energies.

Rani [20] proposed an energy-efficient scheme for the IoT using low power for intracluster communication and high power for intercluster communication. The communication mechanism reduces not only energy consumption for communications but also communication interference among neighboring sensors. We propose that sensors employ different power levels for different targets. The sensors employ low power for checking events with near neighbors and high power for transmitting checked events.

Lekidis [8] assumed that wireless communications increase energy consumption when transmissions collide or interfere with other transmissions. They focused on mechanisms and protocols for low-power wireless communication, as well as on energy-efficient device hardware design. Kovatsh [29] proposed duty cycling of sensors to reduce the amount of transmissions and thus transmission interference and collisions. We propose reducing interference through the appropriate use of smart antennae whilst transmitting or receiving events. Transmission distance may be longer using smart antennae than using omnidirectional antennae, and the range of interference is narrower at the same transmission power.

Reddy [17], Moosavi [21], Marco [22], and Cavalcante [23] proposed saving energy by avoiding duplicate transmissions for event notification. However, duplicate transmissions are sometimes used for verification when some sensors do not detect events due to unpredictable mistakes. Faillettaz [5,6] and Meyer [9] designed codetection mechanisms for monitoring the occurrence of important events. Our proposed mechanism eliminates duplicate transmissions and undetected mistakes by checking events with neighbors before notifying them.

To transmit event information to sinks quickly, Mahmud [19] proposed an energy-efficient transmission routing mechanism. Messages are transmitted following a path from a prearranged directed acyclic graph. When there are two or more sensor nodes that can both be the parent node in a path, the sensor possessing more energy is preferred because it may require energy to help transmit messages. In our proposed mechanism, we also consider residual sensor energy to balance the energy for prolonging the lifetimes of sensor networks. In addition, our mechanism does not decide transmission paths in advance because residual sensor energies are different from transmission situations.

To accommodate monitoring systems to changeable environments, Silva [10] proposed a flexible data gathering protocol that changes monitor attributes according to previous environments and sensor situations. Meyer [9] designed a feedback mechanism to control event notifications flexibly, under which feedback messages are made by a complex decision system according to a large amount of monitoring information. This paper proposes a mechanism that maintains the flexibility to adjust monitoring variables by periodically sharing environment information.

Our mechanism aims to announce events to sinks efficiently and accurately. To ensure events are announced correctly, detected situations are confirmed among neighbors before they are announced. To announce confirmed events efficiently, events are parallel transmitted by a well-designed mechanism in smart antennae. Moreover, to improve the flexibility of the system, the mechanism periodically adjusts the parameters and updates the environment situation.

## 3. Architecture of the Proposed Mechanism

This section introduces the SSRM for WSNs. This mechanism aims to improve the monitoring accuracy and prolong the lifetime of monitoring sensor networks. The main purpose of monitoring sensor networks is to detect abnormal situations (i.e., named anomalies) and notify the sink for driving the actuator and dealing with the anomalies. Because the energy consumption for transmitting is much more than that for sensing, this mechanism proposes to reduce consumption and transmission interference in smart antennae. The sensors do not transmit all sensing data to sinks: they transmit only the values of sensor parameters exceeding a set normal range, which are named as abnormal situations, anomalies, or events. The announcement policy can reduce a large number of transmissions and corresponding energy consumption, particularly when the ratio of anomalies to normal situations is low. In our design, any abnormal situation in the environment will be detected by two or more sensors. If detected, anomalies are mutually confirmed among neighboring sensors. When two or more sensors detect the same anomaly, the anomaly is assumed to be an event and should be transmitted to inform corresponding sinks. When an event is transmitted, transmissions are made with less energy consumption and interference. The details of the mechanism are described below.

### 3.1. Network Environment and Setting

**Figure 1:**
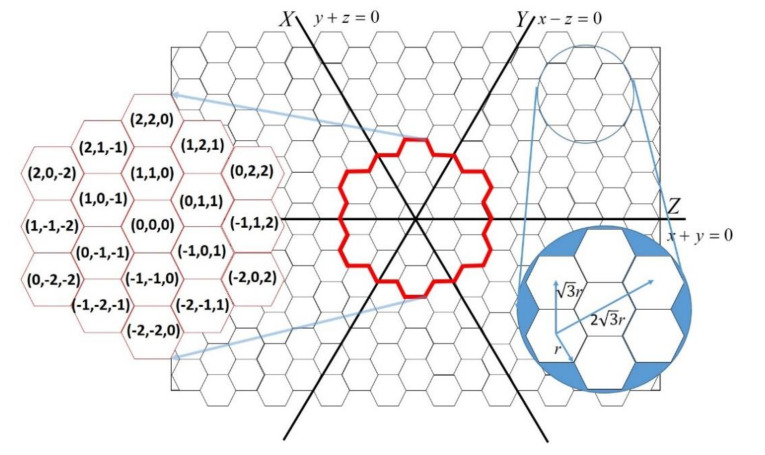
Example coordinates for part of the environment.

The monitored area is divided into *n* equally sized hexagons, hereafter named cells. Each cell has six edges with a length of *r*. In addition, a set of *n* static sensors S = {*s*_1_,*s*_2_,…,*s_n_*} is deployed in the center of these cells. For ease of presentation, we defined a virtual location system with three axes dividing the area into six regions in the monitored environment, where the center of the environment is set by the coordinate (0, 0, 0). The horizontal line crossing the origin (0, 0, 0) is the Z-axis; the Z-axis faces 60° counter-clockwise to the Y-axis, and the Y-axis faces 60° counter-clockwise to the X-axis. The coordinate system is the same as that previously proposed by Hung [13]. The location of each cell is represented by a coordinate (*x*, *y*, *z*). The points to the right of the origin have positive values with regard to the axis, and the distance unit is 3*r*/2. Points on the X-axis, Y-axis, and Z-axis have the same characteristics as *y* + *z* = 0, *x* − *z* = 0, and *x* + *y* = 0, respectively. Figure 1 shows the axes in an environment with some example coordinates.

When monitoring the environment, if any abnormal situation is detected by the sensors, they will notify corresponding sinks of the abnormal situation for them to respond. We assume that the sensors know the locations of corresponding sinks. The entire area of the environment must be monitored by sensors at each period. If the energy of some sensors is exhausted, causing regions to be unmonitored or abnormal situations to go unnotified, then the monitoring network does not work well, and the lifetime of the sensor network can be considered to have ended.

**Figure 2:**
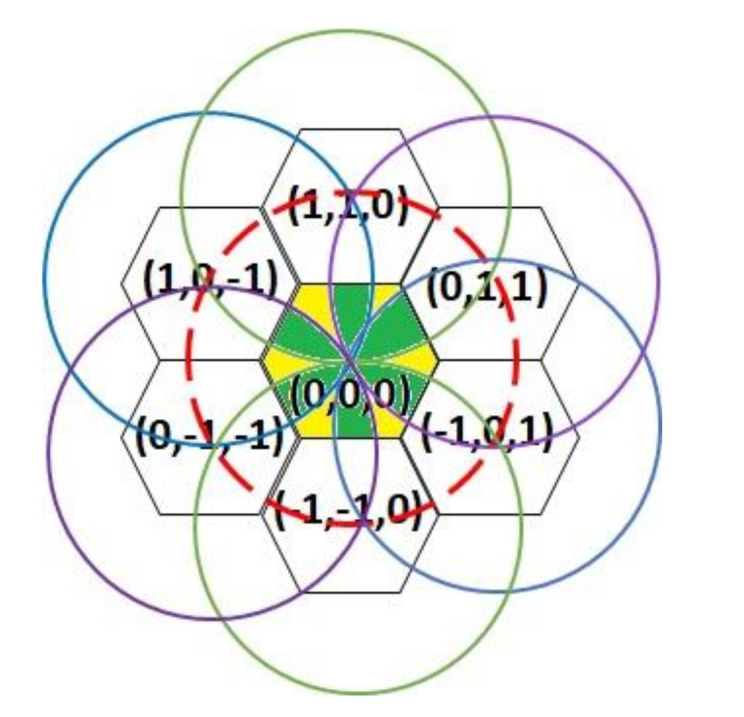
Neighboring sensors monitoring the cell with coordinate (0, 0, 0).

Each sensor is equipped with an omnidirectional antenna and a smart antenna. The omnidirectional antenna is used for sensing and checking events within a distance of $\sqrt{3}r$. Because the sensing range of the sensors is $\sqrt{3}r$, every region in the environment is monitored by three or four sensors. For instance, the region in the cell with coordinate (0, 0, 0) is monitored by the sensors located in coordinates (1, 1, 0), (0, 1, 1), (−1, 0, 1), (−1, −1, 0), (0, −1, −1), and (1, 0, −1). As shown in Figure 2, the regions colored yellow are monitored by three neighboring sensors, and the regions colored green are monitored by four neighboring sensors. The monitoring accuracy is improved by means of mutually checking abnormalities among neighboring sensors in the environment. The transmission distance of the directional antennae is $2\sqrt{3}r$, and these antennae are used to transmit to sensors located in a fan-shaped region at 60° and distance less than $2\sqrt{3}r$ in one of the six directions.

In WSNs, the energy of sensors is limited, and it is consumed by detecting anomalies and transmitting and receiving events. In particular, the energy consumed for transmission is much greater than that for detection and reception. When transmissions collide or interfere with each other, the limited energy of sensors in the monitoring network is wasted. If some sensors exhaust their energy sources, the monitoring system may lose its functionality in monitoring the environment and dealing with problems in time. Therefore, the main objectives of this paper are to improve the accuracy of event detection and prolong the lifetime of the sensor networks monitoring the environment. Let $CM_{i}^{t}$, $CT_{i}^{t}$ and $CR_{i}^{t}$ be the energy consumption for sensing, transmitting and receiving in the *t*th time period, respectively. The initial energy of each sensor is $E^{init}$, which is the amount of total initial energy divided by the number of sensors. The residual energy of sensor *s_i_* at *t*th time period, $E_{i}^{t}$, is obtained by Equation (1). In the proposed mechanism, a region is unmonitored when the region is monitored by less than two sensors. Let ε be the tolerance rate of uninformed events and ω be the acceptable ratio of the unmonitored region to the entire monitoring environment; we would like to maximize the lifetime of environment monitoring subjected to the rate of uninformed events in each round, denoted by $\Phi_{i}$ for the *i*th round, is less than ε and the rate of unmonitored region in each round, denoted by $\sigma_{i}$ for the *i*th round, is less than ω before the monitoring lifetime ends, which is shown as Equation (2)

(1) $$E_{i}^{t}=E_{i}^{t-1}-CM_{i}^{t}-CT_{i}^{t}-CR_{i}^{t},\;E^{init}\geq E_{i}^{t}\geq 0\;for\;\forall \;s_{i}\in S$$

(2) $$\mathrm{Objective}:\;\begin{array}{l} \mathrm{Maximize}\;T=u\times t\\ \mathrm{Subject}\;\mathrm{to}\;:\forall \Phi_{i}<\varepsilon \;\mathrm{for}\;i\leq t\\ \;\;\;\;\;\;\forall \sigma_{i}<\omega \;\mathrm{for}\;i\leq t \end{array}$$

where *u* and *t* are the time duration of each round and the number of rounds, respectively.

### 3.2. Stages

**Figure 3:**
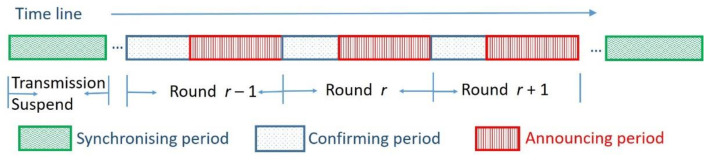
Three types of period in the timeline.

The mechanism proposes that the timeline of a sensor is logically divided into rounds, as displayed in Figure 3. A round consists of a confirming period and announcing period. The lengths of rounds are the same, and the same periods in different rounds are the same length. In both the confirming period and the announcing period, the sensors continue to monitor (sense) the environment. In the confirming period, sensors may communicate with their one-step neighbors within a distance of $\sqrt{3}r$ to check the sensing situation if they detect an anomaly. When sensors confirm the occurrence of an anomaly, a notification event will be transmitted in the announcing period. In the announcing period, events are transmitted forward to corresponding sinks. To transmit events efficiently, the transmissions are arranged so as to prevent interference. Note that if sensors receive or detect events in the confirming period, they cannot transmit events in the following announcing period. Otherwise, the events may be transmitted in the announcing period of the next round. The details of the confirming period and announcing period are described in the following subsections. Because the sensors may clock time at different speeds, they should synchronize periodically. This mechanism proposes that the sensors exchange information summaries and adjust sensing parameters during synchronizing periods. The details of these periods are described in the following subsections.

### 3.3. Communications in the Confirming Period

In our proposed mechanism, the efficiency of this transmission policy depends on accurate anomaly detection, and thus detections should be checked before they are transmitted as events. The confirming period aims to confirm the occurrence of events. To reduce the energy consumed by transmissions, only confirmed events are transmitted to corresponding sinks during announcing periods. The sensor networks do not work well if any events are not notified to the sinks. To improve monitoring accuracy, neighboring sensors communicate to confirm the occurrence of an event. As mentioned, the sensing range is $\sqrt{3}r$, and each cell in the environment has six edges with a length of *r*. The entire environment must be monitored by three or four sensors. If one sensor does not detect abnormal situations for some unexpected reason, two or more sensors will still remain to detect the anomaly. When sensors detect an anomaly, they send an anomaly message to one-step neighbors within a $\sqrt{3}r$ transmitting range using omnidirectional antennae. To avoid messages colliding, the sensors send the anomaly message after a time period *ts* derived by Equation (3), where *D_i,j_* is the distance between a sensor *s_i_* and the nearest corresponding sink *k_j_*; *D_max_* is the longest distance in the environment; *E_i_* and *E^init^* are the residual energy of *s_i_* and the initial energy of each sensor, respectively; and *rt* is the residual time of the current confirming period. If an anomaly was detected in the previous round, the value of *ω* is 2; otherwise, the value of *ω* is 1. That is, an anomaly detected in the previous round has a higher priority than others detected in the current round. Following Equation (3), the sensors a shorter distance away from corresponding sinks and with more energy will have smaller residual time *ts* and will thus send the message earlier. After receiving one or more anomaly warnings, including those detected by themselves, the sensors confirm the occurrence of events. In addition, the sensor which sent the message communicating an event first will transmit the announcement in the following announcing period because it would be the most appropriate one to do so. After communication, other sensors detecting the same anomaly can be certain that the event will not be ignored or missed, because it will be announced by a neighbor.

(3) $$ts=(D_{i,j}/D_{\max})/(E_{i}/E^{init})\times (rt/\omega ),$$

### 3.4. Transmissions in the Announcing Period

In the announcing period, the sensors transmit confirmed events forward to corresponding sinks. In order to reduce transmission steps and energy consumption, each sensor’s transmission distance is $2\sqrt{3}r$. In other words, the neighbors two steps away from the transmitting sensor will receive the message. To avoid interference and reduce energy consumption, the sensors transmit events using smart antennae at 60°, and sensor transmissions during the announcing period are divided into six mini-slots. The sensors in the environment are divided into three groups according to their coordinates, and sensor transmissions are arranged in directions and mini-slots according to their groups. Moreover, in order to avoid interference, sensor receptions are also arranged in directions and mini-slots according to their coordinates. Following this arrangement of transmission and reception, messages will be transmitted to neighbors two steps away without colliding with surrounding sensors.

**Figure 4:**
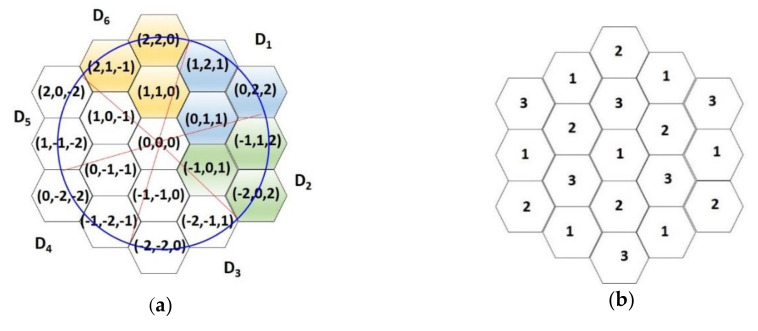
(**a**) Directions and neighbors of the sensor at coordinate (0,0,0); (**b**) sensor groups according to the coordinates of (**a**).

As mentioned, the environment is divided into six regions by the X-axis, Y-axis, and Z-axis. Each cell in the system is denoted by a coordinate (*x*, *y*, *z*) and the cell at the intersection of the three axes is the origin with coordinate (0, 0, 0). For ease of presentation, we named the six directions in which the neighbors of each cell with coordinate (*x*, *y*, *z*) face D_1_ through D_6_. The neighbors in direction D_1_ have coordinates (*x* + *α*, *y* + *β*, *z* + *γ*), where *α* ≥ 0, *β* > 0, *γ* > 0; the neighbors in direction D_2_ have coordinates (*x* + *α*, *y* + *β*, *z* + *γ*), where *α <* 0, *β* ≥ 0, *γ* > 0; the neighbors in direction D_3_ have coordinates (*x* + *α*, *y* + *β*, *z* + *γ*), where *α <* 0, *β <* 0, *γ* ≥ 0; the neighbors in direction D_4_ have coordinates (*x* + *α*, *y* + *β*, *z* + *γ*), where *α* ≤ 0, *β <* 0, *γ* < 0; the neighbors in direction D_5_ have coordinates (*x* + *α*, *y* + *β*, *z* + *γ*), where *α >* 0, *β* ≤ 0, *γ* < 0; the neighbors in direction D_6_ have coordinates (*x* + *α*, *y* + *β*, *z* + *γ*), where *α >* 0, *β >* 0, *γ* ≤ 0. For instance, the neighbors and directions of a sensor at coordinate (0, 0, 0) are depicted in Figure 4a. The neighboring cells of (0, 0, 0) in direction D_1_ are colored blue, the neighboring cells in direction D_2_ are colored green, and the neighboring cells in direction D_6_ are colored yellow. When the antenna direction of sensor s_0_ located at cell (0, 0, 0) faces D_1_, sensors in the blue region can receive information from sensor s_0_, but sensors in the yellow and green regions cannot.

To prevent interference and improve the concurrence of transmissions, the transmitting and receiving of sensors are assigned into three groups, G_1_, G_2_, and G_3_. The sensors are grouped by their coordinates; that is, sensors in different groups have different antenna directions when they transmit events during the same transmission time slot. Each sensor at coordinate (*x*, *y*, *z*) obtains its group by function *F*, *F* = ((*x* + *y*) mod 3). G_1_, G_2_, and G_3_ group sensors have values of *F* being 0, 1, and 2, respectively. For ease of presentation, we have depicted the group number within cells in figures. For instance, if the groups according to the coordinates given in Figure 4a are transformed into Figure 4b, the coordinates (0, 0, 0), (−2, −1, 1), (2, 1, −1), (1, 2, 1), (−1, −2, −1), (1, −1, −2), and (−1, 1, 2) belong to group G_1_, marked as 1; the coordinates (0, 1, 1), (1, 0, −1), (−1, −1, 0), (2, 2, 0), (0, −2, −2), and (−2, 0, 2) belong to group G_2_, marked as 2; the coordinates (1, 1, 0), (0, −1, −1), (−1, 0, 1), (2, 0, −2), (0, 2, 2), and (−2, −2, 0) belong to group G_3_, marked as 3. The sensor transmitting directions are arranged according to groups.

Moreover, sensors can receive messages concurrently using smart antenna arrays. Following the arranged transmission scheme, when a sensor is transmitting in directions D_1_, D_3_, or D_5_, the sensor may receive data from directions D_2_, D_4_, or D_6_; in addition, when a sensor is transmitting in directions D_2_, D_4_, or D_6_, the sensor may receive data from directions D_1_, D_3_, or D_5_. Because the sensor transmission distance is $2\sqrt{3}r$, neighbors within a range of two steps can receive transmissions. In addition, if a neighbor one step away from the transmitting sensor does not close its antenna to that direction, it will receive data intended for its neighbors on the other side. The arranged transmitting and receiving directions are listed in Table 1. For instance, when the 3^rd^ mini-slot, a sensor located in a cell of group G_1_ may transmit its message to its two-step neighbors on direction D_3_ and receive messages from the neighbors on D_6_ and D_2_.

When a sensor receives a message from its neighbors in the first mini-slot, it can relay the message in a later mini-slot if there exists a satisfied direction of transmission. If the directions are not satisfied during later mini-slots, the sensor will relay the message in its mini-slot with a satisfied direction during the next round. For example, for the sensor located in a cell of G_1_, if it receives messages from D_6_ and D_2_ at the 3rd mini-slot, those will be relayed to neighbors on D_2_ and D_5_, respectively. The message to D_5_ can be relayed at the 4th mini-slot in the same round but the message to D_2_ will be relayed at the 2nd mini-slot in the next round.

**Figure 5:**
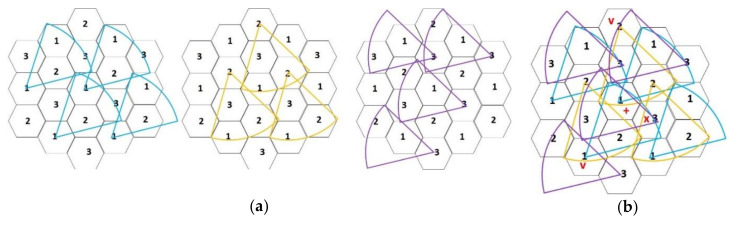
(**a**) Directions of transmissions for sensors located in different groups during the first mini-slot; (**b**) concurrence of transmissions among sensors during the first mini-slot.

For example, Figure 5a shows the directions of transmissions for sensors in groups G_1_, G_2_, and G_3_ during the first mini-slot. In addition, Figure 5b depicts the concurrence of transmissions during the first mini-slot. A sensor *s_i_* in the cell marked with “+” may transmit a message to sensors in D_1_; simultaneously, *s_i_* may receive messages from sensors in D_4_ and D_6_. Thus, *s_i_* can receive messages from the sensors located in cells marked with “∨”. In addition, D_2_, D_3_, and D_5_ are shut off to avoid interference. Because *s_i_* is closed to the direction D_2_, it does not receive the message from the sensor marked “×” that is not intended for it. We can map the cell marked “+” as having coordinates (0, 0, 0). The sensor at (0, 0, 0) may transmit messages to sensors located at (1, 2, 1) and (0, 2, 2) in D_1_ and may receive messages from sensors located at (−1, −2, −1) in D_4_ and (2, 2, 0) in D_6_ during the first mini-slot. Simultaneously, the sensor at (−1, 0, 1) may transmit messages to sensors located at (1, 0, −1) and (0, −1, −1) without interfering with the sensor at (0, 0, 0) because the sensor shuts its antenna to D_2_.

To transmit events efficiently, if the energy levels of two receiving sensors are similar, the sensor that can transmit the event more quickly will forward or relay the event to the sink. Sensors know the transmission directions and mini-slots of their neighbors. They need not negotiate before forwarding. If the difference in energy levels between sensors is large, the sensor that possesses more energy will transmit the event. To ensure that a correct choice is made, energy levels will be exchanged periodically with neighbors as described in the next subsection.

### 3.5. Sharing and Adjusting during the Synchronizing Period

The proposed mechanism arranges the transmitting and receiving times of sensors at different locations. Hence, precise timing among sensors is essential. However, with respect to hardware characteristics, the sensors may clock time at different speeds. Therefore, sensors in the system need to synchronize periodically. The mechanism utilizes a software synchronization technique enabled by information exchange. Moreover, other data may also be shared during synchronization. Shared information includes the residual energy of the sensors, aggregated monitoring data, and monitoring parameters. The synchronization message and monitoring parameters are initiated by the actuator of the monitoring system. The monitoring parameters are used to adjust the system dynamically if required. These parameters include monitoring variable thresholds proposed to be changeable for improved flexibility of the monitoring system. Parameters can be determined by other systems that may include systems for learning knowledge from collected information.

When a sensor catches a synchronizing message with the monitoring parameters and residual energy of a neighbor, it sends out the synchronizing message after removing the residual energy of the neighbor, appending its own residual energy, and aggregating monitoring data from itself. The residual energy of the neighbor is no longer in the message because that information is only intended for one-step neighbors. Neighbors’ residual energies are stored for a sensor to decide whether or not to relay a message when it receives an event announcement from a two-step neighbor in the process described in the final subsection. The aggregated information is the record summarized by each sensor that may be used for realizing the entire system’s situation. The target of aggregated information is the actuator of the system. The mechanism proposes collecting the monitoring record for analyses or predictions. The results of an analysis or prediction may cause the actuator to vary the monitoring parameters.

The flexibility of the proposed mechanism can be achieved by analyzing the information collected in the synchronizing period. The sinks collect the situations of the monitoring environment and the statuses of sensors. In order to maintain the quality of monitoring, the sensor’s sensing range and transmission range may adjust according to the statuses of sensors. When some sensors exhaust their energy, there may exist some coverage holes which are the areas monitored by less than two sensors. After knowing the sensors’ situations, the actuator puts adjustment decisions in synchronization messages. For avoiding the interference among transmissions, the adjustment includes not only the sensing range and transmission range but also the modified virtual coordinates of those hexagonal cells. After adjusting the virtual hexagonal cells, the coordinates and groups of sensors are also changed. Therefore, when a sensor catches the synchronizing message including adjustment information, the sensor enlarges its sensing range, transmission range and has a new coordinate. In order to avoid the interference among the sensors in the same hexagonal cell, the sensors work, i.e., detect or transmit, alternately. In addition, the alternation reduces energy consumption and extends the monitoring lifetime. Moreover, when sensors are not arranged as our assumption—for example, deployed randomly or some unpredictable limits—the virtual coordinates system adjusts flexibly to fit the sensors’ deployment.

Because a sensor’s energy is limited and accuracy may not be ensured, the challenge for monitoring systems is to guarantee the accuracy of monitoring situations and transmit anomalies efficiently for event notifications. The sensing policy of the proposed mechanism is to improve the accuracy of monitored information by using the sensor system itself rather than other supporting systems. The transmitting policy of the proposed mechanism is to reduce energy consumption from transmissions and improve the energy balance of sensors in the network and thus extend the lifetime of the monitoring environment. Furthermore, when the monitoring system has detailed information of the environment, applications can be built or adjusted according to the collected data. To enhance the development of related applications, the mechanism proposes enhancing the flexibility of the parameters in the sensors. The system can dynamically determine the parameters according to the requirements. When the policy of the monitoring system changes, the values of the monitoring parameters can be changed correspondingly. The adjustment can be made periodically when sensors synchronize.

## 4. Evaluation and Discussion

This section evaluates the performance of the proposed mechanism against a multiple level energy adaptive mechanism named MLEACH [24] and an energy-efficient cooperative routing mechanism named EERH [25] in terms of the energy efficiencies of sensors and the WSN. In addition, the performance of the proposed mechanism was also evaluated against EERH and a cooperative detection technique (CoDet) [9] in terms of the monitoring efficiency. MLEACH is a cluster-based transmission mechanism for monitoring systems. MLEACH elects cluster heads according to sensors’ residual energy. These head sensors transmit packets to other clusters with a higher level of amplified energy. EERH is an energy-efficient routing mechanism for WSNs that shares the sensor statuses through piggybacking instead of periodic announcements. Sensors using EERH do not negotiate before transmitting packets, and the time of event transmissions is derived according to the residual energy of sensors and their neighbors, as well as the distance from the sinks. In these simulations, the time duration of one round is set to 2 seconds. The sizes of a checking message and an event packet are 0.2 Kbits and 1 Kbit, respectively. To ensure fair comparisons, the deployments follow the best case of each mechanism, and the total amounts of energy should be identical. After the deployment, the numbers of sensors in EERH and SSRM were 225 and 340, respectively. Therefore, in the simulations, the sensing and transmission distances of EERH were $10\sqrt{2}$ and 20 m, respectively. For SSRM, the distances for sensing abnormalities, checking events, and transmitting events were $10\sqrt{3}$, $10\sqrt{3}$, and $20\sqrt{3}$ m, respectively. In the simulations, the number of sensors was 400 when considering the sensors deployed randomly.

The event detection performance of our mechanism was evaluated according to different event ratios, 10%–40%. Because the sensing ranges of neighboring sensors will overlap in some areas, when an event occurs at some overlapping area, if one of the monitoring sensors fails to detect it, the other sensors monitoring the area may detect it. Hence, we simulated the event detection miss ratio according to different sensor fault rates for EERH, CoDet, and SSRM. CoDet is a mechanism in which the sensor system cooperates with a supporting scheme [9]. CoDet was designed to help confirm the details of events. To avoid energy being consumed by unimportant transmissions, CoDet redesigned sensors to be controlled by the supporting scheme. Only when the supporting system senses an abnormality can the sensor system transmit event notifications.

The parameters in the simulations are listed in Table 2. Sensors know the geographical transmission distances of event packets. The amount of amplified energy is determined by the distance between transmitter and receiver. For example, the amplifier of a sensor consumes energy amounting to 10 nJ × 1000 × 10^2^ to transmit a packet of size 1 Kbits across a distance of 10 m.

**Figure 6:**
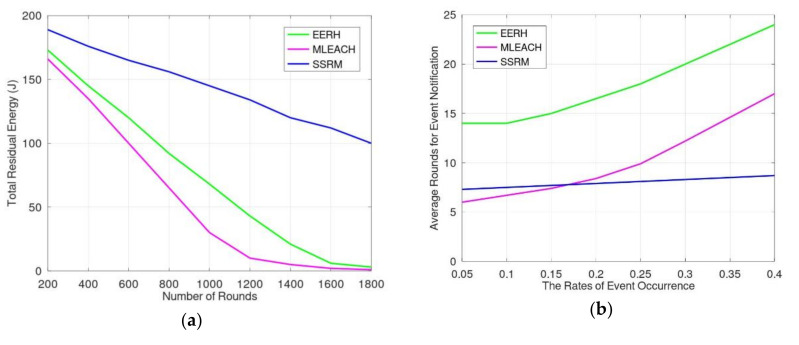
(**a**) Energy consumed by sensors according to monitoring time when the rate of event occurrence is 10%; (**b**) average notification time for one event.

When the event occurrence rate is 10%, Figure 6a shows the results of simulation of energy consumption according to the working time. For a cluster-based scheme, such as MLEACH, the packets are transmitted by less steps due to the long distance of each transmission. On average, because the distance of packet transmission for cluster heads using MLEACH is three times that using EERH and $\sqrt{3}$ times that using SSRM, the steps for event transmission using MLEACH are relatively less. However, the number of interfered sensors for each transmission is relatively more than other mechanisms. Thus, the total energy consumption for transmitting and receiving for MLEACH is more than that for EERH and SSRM. For instance, when the average transmission distance between clusters was 60 m, the average number of interfered sensors in a transmission was 40 sensors. Moreover, MLEACH also consumes energy for status exchange and head election. Therefore, the total residual energy of MLEACH was much less than that of EERH and SSRM when the numbers of rounds were the same as in Figure 6a. Although sensors in SSRM confirm events before transmitting them and they transmit the events across longer distances than those in EERH, the energy consumption for each transmission in SSRM was much less. The reason is that energy is not only consumed for transmissions but is also consumed for reception. According to the SSRM design, sensors adjust smart antennae to some sector region for transmissions and receptions. Thus, the number of influenced sensors is much less. Hence, the energy consumption for transmissions and receptions is relatively less. Furthermore, from the EERH design, redundant event transmissions may occur sometimes. When an event is not transmitted by the sensors located in the shortest path, the event may be announced as redundant in different routes and these sensors may not know the situation. Therefore, according to the above-mentioned reasons, the energy consumption using SSRM is much less than that using EERH and MLEACH. In Figure 6a, when the average residual energy was less than 15mJ, i.e., total energy 3J, many sensors had exhausted their energy. Hence, some events cannot be notified because the routes to sinks were disconnected. Thus, the monitoring lifetime of the WSN ended and the average residual energy of sensors did not decrease obviously.

In addition, we evaluated the efficiency of event notification with different rates of event occurrence, 10–40%. When the rate of event occurrence was less than 0.15, the MLEACH had the highest performance because MLEACH transmits events in a cluster-based model which has the least times of transmission in a fixed distance. However, each transmission in MLEACH influences more sensors than EERH and SSRM, and some transmissions need to wait for other transmissions of neighbors. Thus, as shown in Figure 6b, the rounds for event notifications increased rapidly when the rate of event occurrence was higher than 0.15. Figure 6b shows that the increasing rate of notification time for events in SSRM was much less than that in either MLEACH or EERH. The most important reason is parallel transmissions of mechanisms. Because the number of interfered neighbors is much less in SSRM, the degree of parallelism for SSRM is much higher than others. When the event occurrence rate increased, events could be transmitted to different two-step neighboring sensors simultaneously; thus, the time, i.e., rounds, for these transmissions did not increase obviously. With a lower degree of parallel transmission, the time for event notifications in MLEACH and EERH increased obviously when the event occurrence rate increased.

**Figure 7:**
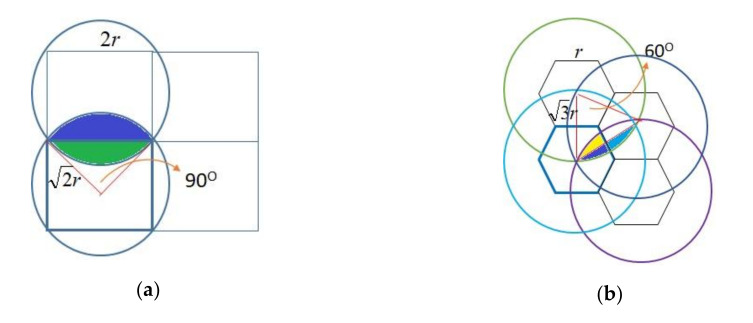
(**a**) An area monitored by two neighboring sensors in mechanisms EERH and CoDet; (**b**) an area monitored by four neighboring sensors in proposed mechanism, SSRM.

Moreover, we evaluated the efficiency of event detection for mechanisms when some sensors faulted. Because the sensing ranges of neighboring sensors may overlap in some areas, the overlapping areas are monitored by two or more sensors which may prevent event misdetections. For instance, when sensors in EERH and CoDet were deployed, as Figure 7a shows, each cell has edges with length 2*r* and the sensing range of each sensor located in the center of cell is $\sqrt{2}r$. As Figure 7a depicts, the overlapped blue region is the same size as the green region, which can be derived from

(4) $$\frac{{\left(\sqrt{2}r\right)}^{2}\pi }{4}-\frac{2r\times r}{2}=(\frac{\pi }{2}-1)\times r^{2}.$$

In each cell, the region monitored by two sensors is calculated as

(5) $$4\times (\frac{\pi }{2}-1)\times r^{2}=2\pi r^{2}-4r^{2}.$$

Hence, the ratio of overlapping region to a cell of EERH and CoDet is 0.57, derived from

(6) $$(2\pi r^{2}-4r^{2})/4r^{2}=\left(\frac{\pi }{2}-1\right)/1\approx 0.57.$$

In addition, the 0.57 overlapping region is monitored by two sensors. When an event occurs at the overlapping region, it is missed if both sensors fault; when an event occurs at the region monitored by one sensor only, it is missed if the sensor faults. Therefore, when the fault rate of each sensor is *δ*, the miss probability of EERH, represented as Θ, is derived as follows.

(7) $$\Theta \;=\;0.57\times \delta^{2}+0.43\times \delta ,$$

CoDet supports another monitoring scheme to detect the entire environment. Thus, the overlapping of CoDet is higher than EERH because each cell is monitored by sensors and the supporting scheme. However, due to the redesign of sensors in CoDet, only when the supporting scheme detects the abnormality can the detected event be transmitted by sensors. When the supporting scheme is faulty, even if many sensors detect the same event, it cannot be notified; when the supporting scheme is correct, the performance of event detection depends on those sensors, which is derived from (7). Therefore, when the fault rate of the supporting scheme is *γ*, the error probability of misdetection in CoDet, represented as Ω, is derived as follows.

(8) Ω=γ+Θ×(1−γ).

Due to the deployment of SSRM, as shown in Figure 1 and Figure 2, Figure 7b depicts that the overlapped blue and yellow region is the same size as the blue and cyan region, which can be derived from

(9) $$\frac{{\left(\sqrt{3}r\right)}^{2}\pi }{6}-\frac{\sqrt{3}r\times 3r/2}{2}=\frac{2\pi -3\sqrt{3}}{4}r^{2}.$$

In each cell, the region monitored by four sensors, the green overlapping region in Figure 2, is obtained by

(10) $$6\times \frac{2\pi -3\sqrt{3}}{4}r^{2}=\frac{6\pi -9\sqrt{3}}{2}r^{2}.$$

Hence, the ratio of the region overlapped by four sensors to the entire cell of SSRM is 0.63, derived from

(11) $$(\frac{6\pi -9\sqrt{3}}{2}r^{2})/(6\times \frac{3\sqrt{3}}{4}r^{2})\approx 0.63,$$

and the ratios of the region overlapped by three sensors to the entire cell is 0.37. According to the SSRM design, when an event occurs at the region monitored by three sensors, the event is missed if only one sensor finds it. In other words, the event is missed when two or more sensors fault. Similarly, when an event occurs at the region monitored by four sensors, it is missed when three or more sensors fault. Therefore, when the fault rate of a sensor is *δ*, the miss probability of SSRM, represented as Φ’, is derived as follows.

(12) $$\Phi ’=0.37\times (3\times (1-\delta )\times \delta^{2}+\delta^{3})+0.63\times (4\times (1-\delta )\times \delta^{3}+\delta^{4}).$$

**Figure 8:**
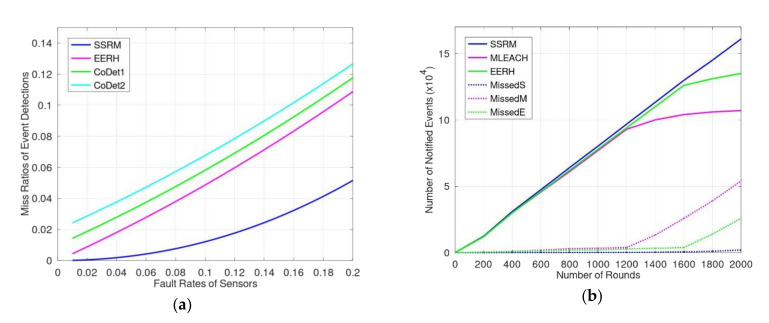
(**a**) The miss rates of event detection for mechanisms with different sensor fault rates; (**b**) monitoring performance of mechanisms.

In general, the values of miss probabilities for mechanisms is Ω > Θ > Φ for any possible *γ* and *δ*. Figure 8a shows the simulation results for miss rates of event detection using EERH, SSRM, and CoDet in two cases of rate *γ*: one is 0.01, named CoDet1 and depicted as a green line, and the other is 0.02, named CoDet2 and depicted as a cyan line. The results show that the more overlapping monitored regions, the fewer miss events. Therefore, SSRM has the lowest miss rate of event detections. Although CoDet has more overlapping areas than EERH, the redesign of sensors means that the event notifications are limited by the correction of supporting scheme.

Figure 8b shows the performance of mechanisms when the event occurrence ratio was 10% and the fault rate of the sensor was 2%. The number of notified events counts the events which arrived at their sinks successfully. The dotted lines are the numbers of miss events using those mechanisms, where a miss event was an event unnotified. For instance, MissedS represented the numbers of miss events for SSRM. When the number of rounds was less than 1000, the performance of these schemes was similar. Their event notification ratios were larger than 95%. When the number of rounds was more than 1200, the number of notified events for MLEACH was less than that of the EERH and SSRM. Because some sensors exhausted their energy, the events routed through these sensors were missed. Given that the rate of miss events ought to less than 5%, the lifetime of MLEACH ended when the number of rounds was greater than 1200 because the miss rates of MLEACH at 1200 and 1400 were 3.9%(=0.382/9.602) and 11%(=1.33/11.33), respectively. Note that the total numbers of events produced for the mechanisms on the same number of rounds, i.e., x-axis, were identical. In addition, due to the duration of the experiments, the numbers of events were accumulated according the numbers of rounds. Thus, the larger the numbers of rounds, the more produced events. Figure 8b shows that when the number of rounds reached 2000, the total numbers of miss events and notified events for SSRM were around $0.02\times 10^{4}$ and $16.1\times 10^{4}$, respectively. The ratio of miss events was still less than 1%, and the monitoring still worked. The key factors are that SSRM maintains high quality of event detection and energy efficiency of event transmission.

Furthermore, we consider that the existence of some coverage holes and an uncovered rate less than ω is acceptable. When some sensors exhaust their energy or when the sensors are deployed randomly, the monitored environment exits some uncovered areas which are named coverage holes. The rate of the uncovered region must be less than the acceptable unmonitored rate, ω, which is defined in (2). Let the rates of the overlapped region detected by *k* sensors be denoted by $\alpha_{k}$, i.e., $\alpha_{0}$ through $\alpha_{4}$ where $\alpha_{0}+\alpha_{1}\leq \omega$. According to the SSRM design, when an event occurs at the region monitored by one or no sensor, the event is missed. When an event occurs at the region monitored by two sensors, the event is missed if any one sensor faults; when an event occurs at the region monitored by three or four sensors, the situation is described in Equation (12). Therefore, when the fault rate of a sensor is δ, the modified miss probability of SSRM, represented as Φ’, is derived as follows.

(13) $$\begin{array}{l} \Phi ’=\alpha_{0}+\alpha_{1}+\alpha_{2}\times (2\delta \times (1-\delta )+\delta^{2})+\alpha_{3}\times (3\delta^{2}\times (1-\delta )+\delta^{3})\\ \;\;+\alpha_{4}\times (4\delta^{3}\times (1-\delta )+\delta^{4}). \end{array}$$

**Figure 9:**
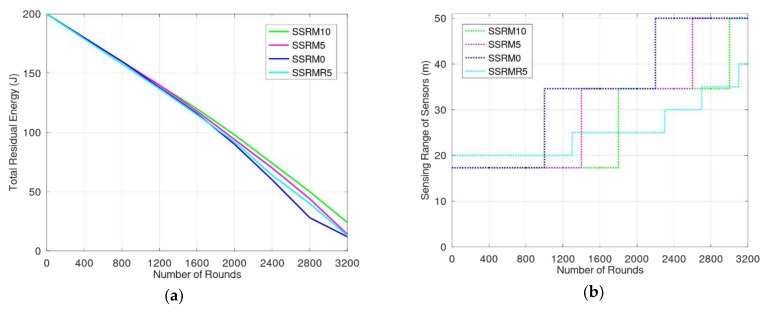
(**a**) Energy consumed by sensors according to monitoring time for different unmonitored region rates; (**b**) sensing range of sensors adjusted in these situations.

If $\alpha_{0}+\alpha_{1}>\omega$, the system will adjust the sensing range, transmission range as well as their coordinates during the synchronizing period. When sensors enlarge their sensing range and transmission range, they consume more energy to detect and transmit events. Figure 9a shows the simulation results for energy consumption of SSRM given different ω when the rate of event occurrence is 25%. SSRM0, SSRM5, and SSRM10 represent SSRM mechanisms with values of ω being 0%, 5%, and 10%, respectively. In addition, SSRMR5 represents the SSRM with ω being 5%, which the sensors deployed randomly. From Figure 9a,b, the residual energy of these mechanisms was similar before 800 rounds because most of the sensors were alive and the sensing ranges of the sensors were the same. The sensing and transmission range of sensors in SSRM0 increased after 800 rounds because some sensors had exhausted their energy and the sensing range was adjusted to achieve full coverage. Because of the enlarging transmission range, the number of interfered sensors also increased. Thus, they consumed more energy than they did before enlarging the sensing range and transmission range. The sensing range of sensors in SSRM10 was the smallest during 1200 and 1600 rounds because SSRM10 allowed for a larger rate of unmonitored region. Note that the sensing range of SSRMR5 is a little larger than others before 800 rounds because the sensors were deployed randomly and the distances among sensors are not as precise as others. Figure 9b shows that the lower the ω, the earlier the sensing range increases to satisfy the acceptable unmonitored rate. The sensing range of sensors in SSRM0, SSRM5, and SSRM10 increased when the numbers of rounds were 1000, 1400, and 1800, respectively. According to the larger sensing range and transmission range, the mechanisms with less ω had less residual energy for the monitoring environment, as shown in Figure 9a.

Moreover, we analyze the flexibility and scalability of the aforementioned mechanisms. As mentioned in previous sections, the sensors periodically share parameters and neighbors’ statuses in synchronizing periods. Thus, the SSRM preserves flexibility by adjusting the sensors’ monitoring parameters. In MLEACH, the sensors communicate their status periodically to elect cluster heads. If the sensor design is modified for adaptive parameters, MLEACH’s degree of flexibility may be achieved by appending the parameters’ values and monitoring records to periodic status communications. Sensors in EERH do not communicate their status periodically. Flexibility can be achieved in EERH similarly to the proposed mechanism by appending the parameter values and monitoring records when synchronizing, except modifying the sensor design. CoDet can achieve flexibility by modifying the supporting system software according to the parameter values. Moreover, aside from sensor statuses and parameters, shared information includes aggregated monitoring data. In these mechanisms, monitored data in normal situations are not announced to sinks to reduce the energy consumed by transmissions. However, monitored information can be used to build related applications. In addition, aggregated data can be used for enhancing the monitoring system such as by gathering statistics that can be used by machine learning or deep learning systems. Therefore, scalability is founded on these monitoring data and related applications.

Furthermore, because the hardware of smart antennae cost much more than omnidirectional antennae, we analyze the performance and consumption for different antennae carefully. For each transmission, a sensor that transmits a packet using a smart antenna to one of its two-step neighbor consumes energy *Ce* to a sector with one-sixth of a circle and the transmitted packet interferes with two neighbors. Following the collision-free arrangement, the sensors in the entire environment can transmit their packets in different directions and a transmission may wait five or less mini-slots for its neighbor’s direction. On the other hand, a sensor that transmits a packet using an omnidirectional antenna to its one-step neighbor consumes energy 6Ce/4 and the transmitted packet interferes with six neighbors. For the collision-free requirement, the two-step neighbors, eighteen neighbors, experience restricted transmission, i.e., let *p* be the parallelism degree of the proposed SSRM using smart antenna, and the parallelism degree using the omnidirectional antenna is *p*×6/2/19 = 3*p*/19. Hence, the energy consumption ratio of using a smart antenna to using an omnidirectional antenna is 4:6; the transmission parallelism ratio of using a smart antenna to using an omnidirectional antenna is 19:1; the transmission performance ratio of using a smart antenna to using an omnidirectional antenna is 19:3. The technique of hardware development progresses quickly, so we assume that the cost ratio of smart antenna to omnidirectional is *s*:*o*. Therefore, when considering the relation between the cost of transmission, cost of hardware, and performance of the antennae, the occasion of using the smart antenna, *Ws*, can be written as follows.

(14) $$Ws=w_{p}(\frac{19}{3})/\left[w_{e}(\frac{4}{6})\times w_{c}(\frac{s}{o}\right)],$$

where *w_p_*, *w_e_*, and *w_c_* are the weighting values of parallelism, energy consumption, and cost of hardware. The larger Ws means that it is more appropriate to use a smart antenna.

To sum up the simulation results, SSRM proposes two or more sensors to monitor the entire environment, so the accuracy of event detections is greater than other mechanisms and the rate of missed events is lower. That is, the monitoring performance of SSRM is better than other mechanisms. These are shown in Figure 8a,b. In addition, because we make good use of smart antennae, the proposed SSRM has greater parallelism and less interference transmissions. The transmission efficiency is much greater than other mechanisms; these are depicted in Figure 6a,b. Moreover, according to the sharing and adjusting of the synchronizing period, the proposed SSRM having the ability to adjust the sensing range and transmission range, the sensors may adapt themselves to varied monitoring environments and to extend the monitoring lifetime; these are shown in Figure 9a,b.

## 5. Conclusions and Future Work

This paper proposes a mechanism for improving the monitoring efficiency and routing efficiency of WSNs. All sensors are equipped with an omnidirectional antenna and a smart antenna for communications and transmissions. By increasing the overlap of regions monitored by sensors, monitoring efficiency is enhanced because sensors confirm event occurrences with neighbors. To avoid transmission interference, the transmissions are arranged among sensors. We designed an energy-efficient and less interfered model for transmission in WSNs.

The results of the evaluations and simulations demonstrate that the miss rate for event detection is much less than for other mechanisms. Because the smart antennae increase parallel transmissions and transmission distances, routing efficiency is enhanced. Following the proposed mechanism, energy consumption is reduced due to reduced communications and faults or duplicate announcements. Therefore, sensor lifetimes are extended. Data records are preserved for future data analysis or artificial intelligence applications. Moreover, because parameter values are changeable for adjusting the monitoring system to conform to the needs of applications, the implementation of intelligence of things or artificial intelligence of things will be easier. Our future work will include building learning mechanisms for WSNs and integrating intelligent monitoring systems.

## Figures and Tables

**Table 1:** Arranged transmission and reception directions for sensors in different groups and different mini-slots. The Tra and Rec represent the transmission action and reception action, respectively.

Group		G_1_		G_2_		G_3_	
	Action	Tra	Rec	Tra	Rec	Tra	Rec
Mini-Slot							
1^st^		D_1_	D_4_&D_6_	D_3_	D_6_&D_2_	D_5_	D_2_&D_4_
2^nd^		D_2_	D_3_&D_5_	D_4_	D_5_&D_1_	D_6_	D_1_&D_3_
3^rd^		D_3_	D_6_&D_2_	D_5_	D_2_&D_4_	D_1_	D_4_&D_6_
4th		D_4_	D_5_&D_1_	D_6_	D_1_&D_3_	D_2_	D_3_&D_5_
5th		D_5_	D_2_&D_4_	D_1_	D_4_&D_6_	D_3_	D_6_&D_2_
6th		D_6_	D_1_&D_3_	D_2_	D_3_&D_5_	D_4_	D_5_&D_1_

**Table 2:** Simulation parameters.

Parameter	Value
Size of network field	300 m × 300 m
Number of deployed sensors	225/340/400
Transmission packet size	1 Kbits/pkt
Mutual checking packet size	0.2 Kbits/pkt
Total initial energy of sensors	200 J
Energy consumed for election	50 nJ/bit
Energy consumed for transmission/receiving	50 nJ/bit
Energy consumed for sensing	30 nJ/s
Energy consumed by the amplifier	10 nJ/bit/m^2^
Event occurrence ratio	10–40%
